# Characterization of Key Aroma Compounds and Main Contributing Amino Acids in Hot-Pressed Oil Prepared from Various Peanut Varieties

**DOI:** 10.3390/molecules29091947

**Published:** 2024-04-24

**Authors:** Jie Sun, Chunhua Zhang, Yu Song, Baijun Chu, Mingqing Wang, Zhiran Zhang, Xiangyu Wang

**Affiliations:** 1 College of Life Sciences, Qingdao University, Qingdao 266071, China; sjj605@163.com (J.S.); zzrzgj@163.com (Z.Z.); 2 COFCO Nutrition & Health Research Institute, Beijing Key Laboratory of Nutrition & Health and Food Safety, Beijing Engineering Laboratory of Geriatric Nutrition Food Research, Beijing 102209, China; zhangchunhua@cofco.com (C.Z.); chubaijun@cofco.com (B.C.); 3 Shandong Peanut Research Institute, Qingdao 266100, China; songyu1103@163.com (Y.S.); wangmingqing2006@126.com (M.W.)

**Keywords:** peanut oil, volatiles, OAVs, pyrazine, correlation

## Abstract

The production of peanut oil in the industrial sector necessitates the utilization of diverse raw materials to generate consistent batches with stable flavor profiles, thereby leading to an increased focus on understanding the correlation between raw materials and flavor characteristics. In this study, sensory evaluations, headspace solid-phase micro-extraction gas chromatography mass spectrometry (HS-SPME-GC-MS), odor activity value (OAV) calculations, and correlation analysis were employed to investigate the flavors and main contributing amino acids of hot-pressed oils derived from different peanut varieties. The results confirmed that the levels of alcohols, aldehydes, and heterocyclic compounds in peanut oil varied among nine different peanut varieties under identical processing conditions. The OAVs of 25 key aroma compounds, such as methylthiol, 3-ethyl-2,5-dimethylpyrazine, and 2,3-glutarone, exceeded a value of 1. The sensory evaluations and flavor content analysis demonstrated that pyrazines significantly influenced the flavor profile of the peanut oil. The concentrations of 11 amino acids showed a strong correlation with the levels of pyrazines. Notably, phenylalanine, lysine, glutamic acid, arginine, and isoleucine demonstrated significant associations with both pyrazine and nut flavors. These findings will provide valuable insights for enhancing the sensory attributes of peanut oil and selecting optimal raw peanuts for its production.

## 1. Introduction

Peanut (*Arachis hypogaea*), which belongs to the legumes, is an important worldwide oil crop valued as the main source of excellent-quality and nutritious aromatic oils. Hot-pressed peanut oil is increasingly preferred by consumers worldwide due to its exceptional flavor and elevated nutritional value [1,2,3]. The global production of peanut oil has witnessed a compound annual growth rate (CAGR) of 1.01%, increasing from 6.18 million tons in 2019 to 6.37 million tons in 2022. Moreover, during the same period, global consumption of peanut oil has experienced a CAGR of 0.96%, rising from 6.19 million tons to 6.37 million tons (USDA FAS, 2023).

The flavor components present in peanut oil contribute to a pleasant sensory experience for consumers and synergistically enhance the overall sensory quality of food when combined with other flavor components. The distinct flavor profile of peanut oil is a result of the combined effects of numerous components, including pyrazines, furans, pyridines, ketones, alcohols, hydrocarbons, aromatics, and others [1,4]. These compounds contribute differently to the overall flavor of peanut oil [5,6,7]. A previous study identified 101 flavor compounds in roasted peanut oil by gas chromatography analysis, mainly including 15 pyrazines, 20 aldehydes, 3 furans, 12 alcohols, and 3 pyrroles [8]. Methylpyrazine, 2,5-dimethylpyrazine, and other pyrazine compounds are the key aroma components responsible for the roasted and nutty aromas of roasted peanut oil [9,10,11]. The formation and composition of these volatile flavors are intricately linked to the chemical reactions that take place during the processing of peanut oil, resulting in diverse volatile flavors in peanut oils produced through different processes [12]. Flavor differences caused by different varieties have been identified among some plant oils such as rapeseed oil, olive oil, and pecan oil [13,14,15]. However, there is a lack of systematic research on the differences in flavors among hot-pressed peanut oils derived from various peanut varieties. 

Researchers are paying more attention to what factors affect hot-pressed peanut oil flavor development. Some research has demonstrated that roasting remarkably improves the quality of peanut oil, especially its flavor and antioxidant activity [16,17]. Furthermore, linoleic acid is regarded as an important precursor for the formation of aroma components of hot-pressed peanut oil. This may be due to the facilitation of the Maillard reaction by lipid-derived free radicals or the promotion of unstable dihydropyrazine compounds through aldehydes during lipid degradation, ultimately leading to the synthesis of long-chain pyrazines [10,12]. Additionally, amino acids are known to play a significant role in the development of diverse flavor compounds in peanut oil. In the Maillard reaction (MR), amino acids undergo complex oxidative degradation, isomerization, and cyclization to form heterocyclic compounds such as pyrazines, pyridines, and pyrroles [18]. Pyrazine compounds correlate significantly with a roasted flavor and aroma. Studies have shown that the condensation of two amino-carbonyls produced by Strecker degradation forms pyrazine compounds during peanut roasting [19]. At this stage, different amino acid precursors have important effects on the formation of flavor substances [20]. Numerous studies have confirmed that adding amino acids such as lysine and arginine can promote the creation of pyrazine flavor substances in Maillard reaction systems composed of amino acids and sugars [21]. Studies have confirmed that volatile precursors such as arginine, tyrosine, lysine, and glucose provide a more potent oil aroma and lead to more characteristic flavor compounds forming during peanut oil processing [10]. Therefore, it is crucial to investigate the correlation between the initial concentration of amino acids in peanut materials and the composition of flavor compounds produced after oil pressing.

**Figure 1:**
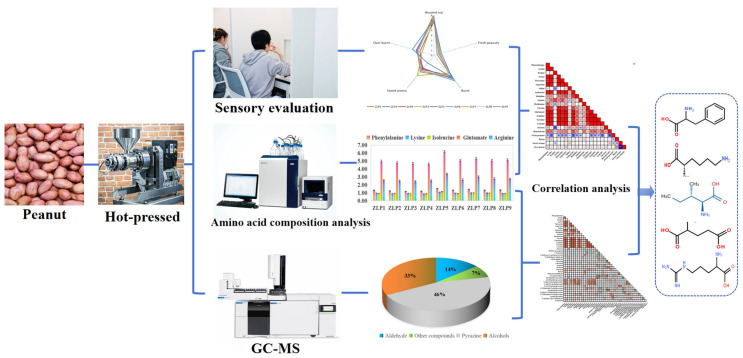
Characterization of key aroma compounds and main contributing amino acids in hot-pressed oil prepared from various peanut varieties.

The variations in the amino acid composition of raw peanut materials have a significant impact on the flavor profiles of peanut oils and can be considered as a potential factor influencing flavors in industrial production. However, there is still a lack of scientific evidence on the major contribution of amino acids to key aroma compounds. Additionally, further research is needed to explore the correlation between the amino acid composition and aroma compounds in different varieties of peanut raw materials. The objective of this study was to conduct sensory analysis and HS-SPME-GC-MS to compare the sensory quality and key aroma compounds of peanut oils derived from nine different varieties. Additionally, we performed physicochemical detection and correlation analysis to examine the relationship between the amino acid composition and flavor composition (Figure 1). These findings will provide valuable data support for raw peanut selection and enhancing sensory attributes during the industrial production of peanut oil. 

## 2. Results and Discussion

### 2.1. Sensory Analysis

**Figure 2:**
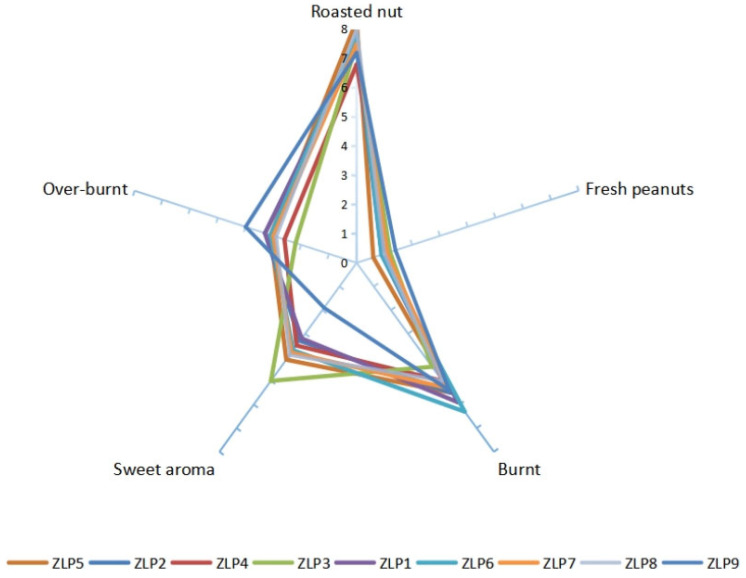
Sensory evaluation results for 9 peanut oil processing samples.

The sensory evaluation results of various samples of peanut oil were represented on a spider web diagram, as depicted in Figure 2. Five sensory indicators, namely, ‘cooked nut flavor’, ‘fresh peanuts flavor’ (peculiar smell), ‘burnt flavor’, ‘sweet flavor’, and ‘paste flavor’ were tested and evaluated. Previous research has shown that the combination of attributes such as ‘roasted nut’, ‘burnt’, ‘sweet aroma’, and ‘over-burnt’ directly influences consumers’ perception of good peanut oil flavor [22]. Under identical processing conditions, ZLP5 exhibited a more pronounced ‘roasted nut’ attribute (8.30), ZLP6 had a stronger presence of the ‘burnt’ characteristic (6.3), ZLP3 possessed a more potent ‘sweet aroma’ attribute (5.0), while both ZLP1 and ZLP3 displayed stronger attributes associated with being ‘over-burnt’ (3.3). The presence of the attribute ‘fresh peanuts flavor’ contributes to an undesirable taste in peanut oil. ZLP9 exhibited a higher level of ‘fresh peanuts flavor’ compared to other samples. Overall, ZLP5 received the highest flavor score, while ZLP4 obtained the lowest score. Based on the sensory evaluations, notable variations in flavor were observed among peanut oils produced from different varieties under identical heating and pressing conditions, indicating that these differences are attributed to variance in the raw materials.

**Figure 3:**
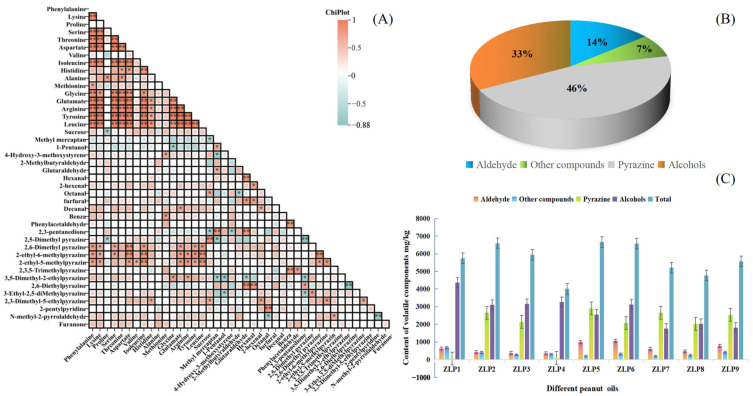
Composition of volatile components in different peanut oils: (**A**) Correlations between amino acids and OAVs of core flavor factors, * *p* < 0.05, ** *p* < 0.01; (**B**) the proportions of different flavor substances in all samples; (**C**) the contents of flavor substances in different samples.

The correlation analysis revealed a negative association between the flavor attributes of fresh peanuts and ‘over-burnt’ and ‘sweet aroma’. Notably, there was a significant negative correlation with ‘sweet aroma’ (*p* < 0.05), indicating that enhancing these two flavors can help eliminate the peculiar odor of peanut oil (Figure 3A). Furthermore, a strong negative relationship was observed between the flavors of ‘sweet’ and ‘over-burnt’ (*p* < 0.01), suggesting that reducing the ‘over-burnt’ flavor can enhance the sweetness flavors of peanut oil.

### 2.2. Volatile Components of Different Peanut Oils

After the analysis of nine peanut oil samples using HS-SPME-GC-MS, a total of 125 aroma compounds were identified in the samples (Table S1). These compounds included 22 aldehydes, 45 heterocyclics, 9 alcohols, 13 alkanes, 6 acids, 13 ketones, 9 esters, 2 phenolics, and 7 others. Among them, heterocyclic compounds (including 18 pyrazines) exhibited the highest volatile component contents, followed by aldehydes and alcohols (Figure 3B). Heterocyclic compounds, aldehydes, and alcohols are crucial constituents of volatile flavor components in peanut oil [16]. Pyrazines, such as 2,5-dimethylpyrazine and 2-ethyl-3-methylpyrazine, predominantly contribute to the roasted nut aroma that imparts a strong roasted scent to peanut oil. Conversely, aldehydes enhance the pleasant taste of peanut oil by adding oily and sweet flavors. Additionally, certain ketones and alcohols can impart fruity and vegetal aromas. Collectively, these flavor components synergistically contribute to the overall olfactory profile of peanut oil [6]. 

Based on the findings, ZLP5 exhibited higher concentrations of aroma compounds compared to other peanut oil samples (Figure 3C). In contrast, ZLP4 had the lowest amounts. The content of alcohols was highest in ZLP1 and lowest in ZLP7. These findings are consistent with the sensory evaluations, as ZLP5 demonstrated the highest content of aroma compounds, along with receiving the highest sensory evaluation score. Notably, ZLP5 displayed the greatest pyrazine content and achieved the highest roasted nut score during sensory evaluations. Meanwhile, ZLP6 showcased a wider range of aldehydes and received the highest ‘burnt’ score. These findings demonstrate that different varieties of peanuts processed under identical conditions yield distinct aroma compounds in their resulting oils, thereby giving rise to diverse flavor profiles.

The flavor profiles of peanut oil produced under identical processing conditions in various African peanut varieties yielded similar outcomes. Six samples of Malawi peanut varieties were subjected to roasting, and the volatile components were detected using HS-SPME-GC-MS. Notably, two peanut varieties (Nsinjiro, Baka) exhibited a significantly higher intensity of roasted peanutty flavor (*p* < 0.05) [23]. These findings highlight the significant challenges faced by peanut oil companies in achieving consistent flavors due to variations in production material availability.

### 2.3. OAVs of Key Aroma-Active Compounds

The main odorants of hot-pressed peanut oils have been reported to include aldehydes, pyrazines, alcohols, and other heterocyclic compounds [16]. However, not all flavor compounds equally impact the overall aroma perception of peanut oils. Only volatile compounds present in concentrations that surpass their respective odor thresholds can be detected by humans [1]. Both the concentration and odor threshold of a volatile compound play crucial roles in shaping the overall flavor characteristics of peanut oils. Consequently, the odor activity values (OAVs) were calculated for volatiles in peanut oils by dividing their concentrations by their corresponding odor thresholds in oil. Volatile compounds with OAVs greater than 1 are considered significant contributors to the overall flavor profile of peanut oil. The OAVs for key active odor constituents across different samples are presented in Table 1.

The nine samples contained a total of 125 active odors, out of which 25 had odor activity values (OAVs) above 1, indicating their significant contribution to the aroma profile of peanut oil. Among all the samples, methyl mercaptan (methanethiol) exhibited the highest OAV of 4350 in ZLP1, followed by 3-ethyl-2,5-dimethylpyrazine, with an OAV of 2742 in ZLP5. Additionally, 2,3-pentanedione showed an OAV of 670 in ZLP1, while 2-methylbutyraldehyde and phenylacetaldehyde had OAVs of 354.5 and 293.75 in ZLP1 and ZLP5, respectively. These aromatic substances collectively enhanced the rich flavor characteristics observed in peanut oil. Moreover, other flavor compounds with lower but still significant OAV values (>1) also played a role in shaping the overall flavor profile. 

Methanethiol is a crucial aroma compound in peanut oil and significantly contributes to its overall aroma profile. We observed the highest concentration of methanethiol in ZLP1 peanut oil, while the lowest was found in ZLP7. Chetschik et al. reported that methanethiol had the highest odor activity value (OAV) among roasted peanut meal compounds. Stable isotope dilution assays (SIDAs) and aroma reconstitution experiments demonstrated the significant role of methanethiol in shaping the overall aroma characteristics of roasted peanuts [24].

The compound 3-ethyl-2,5-dimethyl pyrazine exhibits a toasted nut aroma. Being one of the most crucial pyrazine aroma compounds, it has previously been identified as a key odorant in roasted peanut oils [4,25]. In this study, we observed its highest concentration in ZLP5; its concentration was slightly lower in the other samples, and it was completely absent in ZLP4. Pyrazines with characteristic roasted/nutty flavors were found to be the predominant odorants in the roasted peanut oil. A total of 18 pyrazine odorants were detected, out of which 12 had OAVs exceeding 1. The OAVs of these pyrazine odorants varied significantly among different samples, possibly due to variations in raw material flavor profiles. Additionally, other pyrazine odorants such as 2,5-dimethylpyrazine and 2,6-dimethylpyrazine exhibited high concentrations in ZLP5, contributing to its highest sensory score.

We also observed that the critical ketones and aldehydes (2,3-pentanedione, 2-methylbutanal, and phenylacetaldehyde) present in peanut oil exhibited higher OAVs across different samples. Previous studies have identified 2,3-pentanedione as a characteristic Maillard reaction product contributing to the buttery aroma of roasted peanut oil [1,26]. Chetschik further demonstrated that both 2-methylbutanal and phenylacetaldehyde were formed through amino acid degradation during the peanut roasting process [24]. These compounds possess distinct sweet aromas and significantly contribute to the overall flavor profile of peanut oil [27].

### 2.4. Correlation between Amino Acids and Sensory Evaluation Attributes in Different Samples

Proteins and sugars are considered the primary precursors for aroma development in peanut oils. In recent years, there has been significant interest in the reactivity of amino acids in Maillard reactions [28]. The process of roasting peanuts leads to the formation of pyrazines, furans, pyridines, and other aromatic compounds through Maillard reactions, Strecker degradation, and caramelization [16]. Newell and Mason distinguished between amino acid precursors associated with typical roasted peanut flavor production and those associated with atypical roasted peanut flavor [29]. The typical precursors of roasted peanut flavor include aspartic acid, glutamic acid, glutamine, asparagine, histidine, and phenylalanine, while threonine, tyrosine, lysine, and arginine are also involved [30]. A pathway analysis revealed that compounds associated with amino acid metabolism underwent the most changes during peanut oil production [31]. Pattee et al. discovered a positive correlation between total sugars and roasted peanut aromas [32]. Therefore, differences in Maillard precursor concentrations significantly impact the flavor of peanut oil.

**Figure 4:**
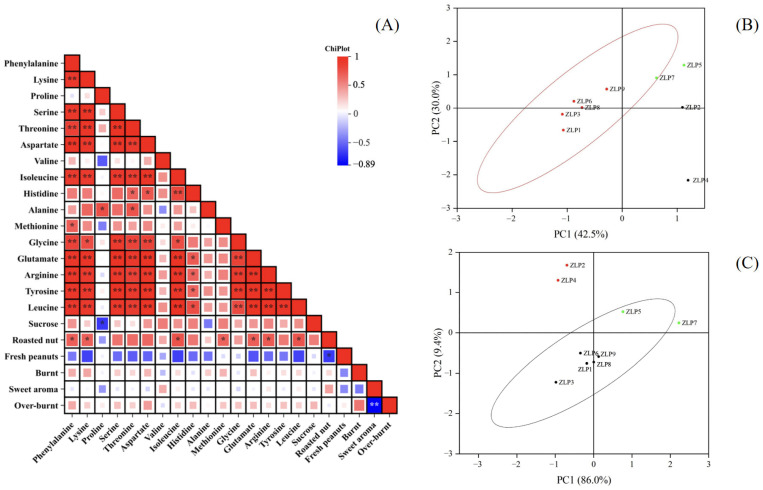
Correlations between amino acids and sensory evaluations of oil processing samples and a principal component analysis (PCA) score chart. (**A**) Correlations between amino acids and OAVs of core flavor factors, * *p* < 0.05, ** *p* < 0.01; (**B**) PCA of pyrazines in different samples; (**C**) PCA of pyrazines related to amino acids in different samples.

We conducted an analysis on the fatty acid compositions, amino acids, and total sugar contents of nine peanut samples in this study. The oleic acid contents in these peanut oils were all found to be below 70%, indicating that they were normal-oleic peanut oils (Table S2). The amino acid contents of different samples are presented in Table 2. Duncan’s analysis revealed significant variations in the contents of certain amino acids among some samples (*p* < 0.05). Notably, ZLP5 exhibited significantly distinct levels of precursor amino acids (aspartic acid, glutamic acid, and histidine), as well as atypical amino acids (threonine, tyrosine, and arginine), compared to other samples. The levels of these flavor-related amino acids in ZLP5 were significantly higher compared to those in other samples. Correlations between the amino acid and sucrose contents of various samples, as well as the sensory evaluation attributes, were analyzed, and the results are presented in Figure 4A. The correlation analysis revealed that phenylalanine, lysine, isoleucine, methionine, leucine, glutamic acid, and arginine exhibited significant correlations with the flavor attribute of ‘roasted nut’ (*p* < 0.05). The roasted nut flavor primarily originates from pyrazines.

### 2.5. Correlations between Amino Acids and Core Flavor Compounds

Correlations between the amino acid and sucrose contents of various samples, as well as the 25 selected core flavor compounds (OAVs > 1), were analyzed, and the results are presented in Figure 4. The correlation analysis revealed a significant association between pyrazines and phenylalanine, lysine, glutamic acid, arginine, tyrosine, aspartic acid, phenylalanine, and isoleucine (Figure 3A). Furthermore, the levels of phenylalanine, aspartic acid, lysine, leucine, isoleucine, glutamic acid, arginine, and tyrosine were found to be positively correlated with the contents of 2-ethyl-6-methylpyrazine, 2-ethyl-5-methylpyrazin, and 2,6-dimethylhydrazine (*p* < 0.05). The findings suggest that the presence and alterations in the composition of these amino acids significantly influence the formation of pyrazine flavor compounds. 

Many researchers have extensively investigated the formation pathway of heterocyclic pyrazine compounds, which are known for their characteristic roasted aroma in food [33]. Zou et al. discovered a high Maillard reactivity in the N-terminal dipeptide containing Leu [34]. Similarly, lysine has been demonstrated to contribute to pyrazine formation in the lysine and glucose MR system [35]. Another study confirmed that the bitter-tasting compounds synthesized in the carbohydrate and proline MR system were 7-acetyl-5-methyl-2,3-dihydro-1H-pyrrolizine (7-AMDP) and 5-acetyl-6-methyl-2,3-dihydro-1H-pyrrolizine (5-AMDP) [36]. The levels of aspartate and leucine, which play a crucial role in the formation of key pyrazine compounds in Congou black tea, were observed to decrease during the roasting process [37]. Cuci et al. constructed a Maillard reaction system using xylose, phenylalanine, and cysteine to synthesize various pyrazines [38]. The obtained results are in line with the findings of this study, suggesting a significant involvement of specific amino acids in the formation of pyrazine compounds. Furthermore, the correlation analysis between amino acids and flavor substances, as well as the sensory evaluation properties, revealed that phenylalanine, lysine, glutamic acid, arginine, and isoleucine exhibit associations with both pyrazine and nut flavors. However, further investigation is necessary to fully comprehend this relationship.

The present study also revealed an association between the production of methyl furfural and the presence of serine and glutamate. Methionine correlated with 2-acetylpyrrole and 2-pyrrolphosphate. These findings are consistent with previous research conducted in other studies. Using multivariate statistical techniques, the formation of 5-hydroxymethylfurfural was positively correlated with serine and glutamic acid in coffee beans [39,40]. However, further investigation is warranted to elucidate the impact of methionine, serine, and glutamate on both the production and content of these flavor compounds in peanut oil. Additionally, there was a significant correlation observed between the total sugar content and pyrazines, as well as aldehydes, suggesting that sugars play a substantial role in peanut oil flavor formation.

### 2.6. Principal Component Analysis

We conducted principal component analysis (PCA) on the data matrices of pyrazine odor volatiles in various peanut oils (Figure 4B). PCA effectively distinguished samples ZLP1, ZLP3, ZLP6, ZLP8, and ZLP9 from ZLP5, ZLP7, ZLP2, and ZLP4. Similar results were also obtained from PCA analysis using the data matrices of the 11 pyrazine-related amino acid contents in different peanut materials (Figure 4C). These findings demonstrate that the variation in the raw material composition of the 11 pyrazine-related amino acids is consistent with the differences in pyrazine flavor observed in peanut oil. These amino acids play a crucial role in the synthesis of pyrazines. In future investigations, it is imperative to focus on the specific involvement of these amino acids in the production process of pyrazine compounds and to examine alterations in the generated pyrazine compounds under varying heating temperatures, durations, and other production conditions.

## 3. Materials and Methods

### 3.1. Materials

Nine different peanut varieties were gathered by Cofco Nutrition and Health Research Institute Co., Ltd. (Beijing, China), from different peanut-producing areas in China. The raw peanut sample was roasted at 160 °C for 25 min. Then, the roasted peanuts were pressed at 120 °C to produce peanut oil. Peanut oil samples were filtered to remove impurities and stored at −20 °C. All reagent-grade chemicals were purchased from National Chemical Reagent Co., Ltd., Beijing, China, and Sigma-Aldrich, St. Louis, MO, USA, including methanol, NaOH, HCl, 2-octanol, etc.

### 3.2. Sensory Analysis

A descriptive sensory analysis was used to analyze the odor constituents of the peanut oils. Seven well-trained panelists (three males and four females, aged 25 to 40) from the Laboratory of Flavor Chemistry, Cofco Nutrition and Health Research Institute Co., performed the sensory analysis in a sensory room under the conditions of set temperature and humidity. Details on the methods, lexicon, and attribute definitions followed those previously published by Lykomitros [22]. The panelists determined a list of five descriptive terms for peanut oil by consensus. The sensory attributes used were ‘roasted nut’, ‘fresh peanuts’, ‘sweet aroma’, ‘burnt’, and ‘over-burnt’, and a nine-point scale was used (1 = very weak, 9 = very strong). Table 3 presents descriptions of the flavor sensory attributes. The sensory analysis results are shown in a spider diagram.

### 3.3. Amino Acid Profile Analysis

The amino acid composition and content were determined following the method described by Hu et al. [10]. Amino acid profiling was conducted using an amino acid analyzer. Briefly, a 100 mg sample was hydrolyzed at 110 °C for 24 h in an oven with 10 mL of 6 N HCl containing 0.1% phenol, followed by cooling on ice. Subsequently, 30 mL of citrate buffer (pH 2.2) was added to the bottles while continuously stirring on ice. Additionally, 1 mL of a 500 μM norleucine solution in citrate buffer (pH 2.2) was added to each bottle. The final volume of the solution was adjusted to 100 mL with citrate buffer at pH 2.2 in a volumetric flask. This solution was then filtered and analyzed using Biochrom Limited’s Biochrom^®^20 plus amino acid analyzer (Biochrom Ltd, Biochrom 20, UK).

### 3.4. Total Sugar Content Analysis

Peanut raw materials were skinned manually and pressed to obtain peanut oil and to prepare partially nonfat peanut meals. We stored the peanut oils at −20 °C until fatty acid composition analysis. The partially nonfat meals were further degreased with petroleum ether to prepare completely defatted flours. To analyze the fatty acid composition, we followed the method reported by Lykomitros et al. [41].

### 3.5. Headspace Solid-Phase Micro-Extraction (HS-SPME)

HS-SPME was used to analyze the flavor compounds in nine peanut oil samples. The flavor compounds of peanut oil were extracted using a published method with slight modifications [42]. Flavor compounds were extracted by a 50/30 μm 57328-U SPME fiber (Stableflex, Supelco Co., Bellefonte, PA, USA). Before use, the fiber was pretreated for 60 min at 270 °C. Peanut oil samples (5 g) were placed in a 20 mL headspace bottle which was sealed with an aluminum cover and a Teflon septum. A 25 μL aliquot of 2-octanol (0.25 mg/mL in methanol) was added as an internal standard. After equilibration for 20 min at 55 °C, the SPME fiber was inserted into the bottle and exposed to the headspace for 40 min at 60 °C. The compounds absorbed by the fiber were thermally desorbed via GC for 300 s at 250 °C.

### 3.6. Identification and Quantification of Flavor Components

Volatiles extracted from peanut oil samples were analyzed using a GC system (Agilent 7890B, Agilent Technologies, Santa Clara, CA, USA) and MS detector (Agilent 5977B) equipped with a VF-WAX column (30 m × 0.25 mm × 0.25 μm Agilent CP9205). To identify flavor components, the peanut oils were analyzed by GC-MS using the detailed determination method mentioned by Xu [7]. We identified flavor compounds using the retention times of the n-alkanes C7-C30 by linear interpolation and compared them to the RI of standard compounds in the NIST17 library. Semiquantification was performed for the identified compounds, and the relative concentration was calculated based on the area of the 2-octanol.

### 3.7. Odor Activity Values

The OAVs were used to analyze the contributions of volatiles to the overall flavor of different peanut oils. A flavor compound’s OAV was calculated as the ratio of its concentration in oil to its odor threshold in water, which was obtained from the literature [43].

### 3.8. Statistical Analysis

All experiments were performed three times, and multiple comparisons between the samples were performed via the least significant difference (LSD) method (*p* < 0.05). Significant differences between mean values were determined by the least significant difference (LSD) method. For statistical analysis, the software SPSS was used (IBM SPSS 22.0, Chicago, IL, USA), and we set the confidence level at *p* < 0.05. The correlation heatmap was drawn by the software Chiplot (https://www.chiplot.online/#Heatmap, accessed on 13 March 2024).

## 4. Conclusions

Sensory evaluations of peanut oil produced from nine different peanut varieties under identical processing conditions revealed variations in their sensory attributes. The burnt and sweet aroma characteristics of the oils exhibited a negative correlation with the off-odor properties of fresh peanuts. GC-MS analysis unveiled disparities in the composition and content of aroma components, including alcohols, aldehydes, and heterocycles. Among these components, ZLP5 displayed the highest pyrazine content and the highest sensory evaluation score, indicating that the pyrazine content significantly influenced the overall aroma profile of the peanut oil. Out of the 18 detected pyrazine flavor compounds, 12 had odor activity values (OAVs) exceeding 1. The nine peanut varieties exhibited distinct differences in their amino acid composition, and a correlation analysis with key flavor compounds revealed that 11 amino acids displayed a positive correlation with pyrazines. These findings can provide valuable theoretical insights for the selection of raw materials and the enhancement of oil flavor in peanut oil production. However, further investigation is required to elucidate the specific mechanism underlying the involvement of these amino acids in pyrazine synthesis.

## Figures and Tables

**Table 1:** Odor activity values (OAVs) of key aroma-active compounds in different peanut oils.

		Odorants	Odor Threshold mg/L	OAVs								
				ZLP1	ZLP2	ZLP3	ZLP4	ZLP5	ZLP6	ZLP7	ZLP8	ZLP9
1	Alcohols	Methyl mercaptan	0.00006	4350.00	3200.000	3150.000	3250.00	2550.000	3100.000	1750.000	2000.000	1800.000
2		1-Pentanol	0.15020	3.45539	2.47670	0.00000	2.83622	0.00000	2.69640	0.00000	2.03728	0.00000
3	Phenolics	4-Hydroxy-3-methoxystyrene	0.05000	5.22000	5.94000	7.44000	6.18000	11.10000	15.60000	11.88000	17.34000	15.18000
4	Aldehydes	2-Methylbutyraldehyde	0.00220	354.54545	216.81818	263.18182	197.72727	261.81818	246.81818	278.18182	162.27273	173.18182
5		Glutaraldehyde	0.00900	32.33333	24.33333	14.00000	13.33333	18.00000	13.66667	12.33333	11.00000	17.33333
6		Hexanal	0.27600	4.57609	4.76087	2.41304	2.11957	3.71739	3.20652	2.18478	2.96739	3.42391
7		2-Hexenal	0.08870	0.94701	1.35287	0.50733	0.33822	1.42052	1.11612	0.57497	0.54115	1.38670
8		Octanal	0.05150	1.92233	5.76699	3.26214	5.82524	6.64078	5.82524	5.41748	6.58252	15.20388
9		Furfural	0.97000	1.68557	1.59278	1.35155	0.94330	1.67010	1.08866	0.99588	0.93402	1.05464
10		Decanal	0.00010	0.00000	0.00000	0.00000	0.00000	570.0000	480.0000	0.00000	0.00000	360.0000
11		Benza	0.04170	13.52518	4.38849	6.61871	6.76259	10.07194	17.26619	17.91367	12.30216	12.58993
12		Phenylacetaldehyde	0.00400	219.00000	175.5000	84.75000	123.750	117.7500	293.2500	282.0000	273.7500	195.7500
13	Ketones	2,3-Pentanedione	0.00030	670.000	320.000	260.000	290.000	180.000	150.000	180.000	220.000	90.000
14	Pyrazines	2,5-Dimethyl pyrazine	1.00000	0.00000	4.56300	2.95200	3.51300	5.02800	4.31100	4.85700	4.13100	4.19100
15		2,6-Dimethyl pyrazine	0.71800	1.64206	1.52089	0.93175	0.96100	1.79248	1.20334	1.65042	1.16992	1.33705
16		2-Ethyl-6-methylpyrazine	0.04000	23.47500	27.30000	17.55000	15.52500	34.42500	18.30000	30.97500	16.65000	22.57500
17		2-Ethyl-5-methylpyrazin	0.10000	20.79000	26.16000	17.79000	20.64000	32.46000	25.59000	29.22000	22.08000	18.36000
18		2,3,5-Trimethylpyrazine	1.50000	1.00400	0.00000	0.00000	0.64800	0.00000	0.89000	1.32200	0.84400	1.02800
19		3,5-Dimethyl-2-ethylpyrazine	0.00220	0.00000	0.00000	53.18182	50.45455	90.00000	57.27273	83.18182	40.90909	75.00000
20		2,6-Diethylpyrazine	0.00600	16.00000	20.50000	0.00000	0.00000	0.00000	0.00000	0.00000	0.00000	0.00000
21		3-Ethyl-2,5-dimethylpyrazine	0.00100	0.00000	2670.000	2040.000	0.00000	2742.000	1965.000	2502.000	1941.000	2418.000
22		2,3-Dimethyl-5-ethylpyrazine	0.53000	0.99623	1.52264	0.95660	0.95660	1.46038	1.04151	1.31887	1.06981	1.50000
23		2-Pentylpyridine	0.00060	0.00000	70.00000	0.00000	0.00000	0.00000	140.0000	0.00000	0.00000	315.0000
24	Other heterocyclics	N-methyl-2-pyrrolaldehyde	0.03700	1.05405	0.72973	0.64865	0.72973	1.21622	0.64865	1.21622	0.72973	0.00000
25		Furanone	0.02230	24.08072	18.02691	14.12556	14.66368	20.98655	19.91031	20.44843	26.23318	17.48879

**Table 2:** Amino acid profiles of different peanut oil processing samples (g/100 g).

	Phenylalanine	Lysine	Proline	Serine	Threonine	Aspartate	Valine	Isoleucine	Histidine	Alanine	Methionine	Glycine	Glutamate	Arginine	Tyrosine	Leucine	Total sugar
ZLP1	1.32 ± 0.11 ^abc^	0.95 ± 0.03 ^a^	0.96 ± 0.03 ^a^	1.19 ± 0.28 ^ab^	0.66 ± 0.05 ^ab^	3.06 ± 0.28 ^ab^	1.08 ± 0.05 ^b^	0.92 ± 0.05 ^b^	0.94 ± 0.07 ^ab^	0.95 ± 0.03 ^ab^	0.19 ± 0.05 ^a^	1.42 ± 0.23 ^bc^	4.940 ± 0.13 ^bc^	2.55 ± 0.23 ^bc^	1.02 ± 0.15 ^bc^	1.68 ± 0.21 ^b^	4.8 ± 0.15 ^c^
ZLP2	1.22 ± 0.15 ^bc^	0.88 ± 0.07 ^a^	0.72 ± 0.01 ^bc^	1.08 ± 0.07 ^b^	0.6 ± 0.03 ^b^	2.96 ± 0.27 ^b^	1.54 ± 0.15 ^a^	0.92 ± 0.09 ^b^	1.03 ± 0.03 ^ab^	0.74 ± 0.07 ^cd^	0.17 ± 0.01 ^a^	1.310 ± 0.13 ^c^	4.76 ± 0.28 ^bc^	2.5 ± 0.15 ^bc^	0.95 ± 0.05 ^c^	1.68 ± 0.05 ^b^	4.9 ± 0.36 ^bc^
ZLP3	1.16 ± 0.07 ^c^	0.9 ± 0.07 ^a^	0.94 ± 0.05 ^a^	1.12 ± 0.15 ^ab^	0.62 ± 0.06 ^b^	2.8 ± 0.17 ^b^	0.9 ± 0.03 ^b^	0.85 ± 0.07 ^b^	0.9 ± 0.05 ^ab^	0.86 ± 0.09 ^abc^	0.16 ± 0.01 ^a^	1.4 ± 0.21 ^bc^	4.66 ± 0.35 ^c^	2.44 ± 0.17 ^c^	0.96 ± 0.05 ^c^	1.56 ± 0.09 ^b^	4.8 ± 0.09 ^c^
ZLP4	1.2 ± 0.21 ^bc^	0.85 ± 0.01 ^a^	0.68 ± 0.01 ^c^	1.08 ± 0.03 ^b^	0.6 ± 0.06 ^b^	2.8 ± 0.28 ^b^	1.48 ± 0.06 ^a^	0.87 ± 0.06 ^b^	0.93 ± 0.06 ^ab^	0.710 ± 0.09 ^d^	0.19 ± 0.07 ^a^	1.39 ± 0.07 ^bc^	4.6 ± 0.31 ^bc^	2.54 ± 0.23 ^bc^	0.94 ± 0.08 ^c^	1.6 ± 0.09 ^b^	4.2 ± 0.31 ^c^
ZLP5	1.520 ± 0.13 ^a^	1.08 ± 0.28 ^a^	0.82 ± 0.07 ^b^	1.37 ± 0.03 ^a^	0.73 ± 0.01 ^a^	3.48 ± 0.36 ^a^	1.5 ± 0.28 ^a^	1.13 ± 0.21 ^a^	1.14 ± 0.15 ^ab^	0.92 ± 0.17 ^a^	0.2 ± 0.03 ^a^	1.65 ± 0.15 ^a^	6.16 ± 0.43 ^a^	3.38 ± 0.59 ^a^	1.26 ± 0.21 ^a^	2.1 ± 0.31 ^a^	5.3 ± 0.45 ^ab^
ZLP6	1.32 ± 0.08 ^abc^	0.95 ± 0.01 ^a^	0.65 ± 0.01 ^c^	1.14 ± 0.17 ^ab^	0.62 ± 0.03 ^b^	2.97 ± 0.21 ^b^	1.1 ± 0.07 ^b^	0.9 ± 0.03 ^b^	0.88 ± 0.07 ^b^	0.77 ± 0.06 ^abc^	0.21 ± 0.07 ^a^	1.37 ± 0.17 ^bc^	5.02 ± 0.17 ^bc^	2.64 ± 0.21 ^bc^	0.98 ± 0.09 ^c^	1.67 ± 0.15 ^b^	4.6 ± 0.31 ^c^
ZLP7	1.32 ± 0.03 ^abc^	0.96 ± 0.01 ^a^	0.81 ± 0.03 ^b^	1.18 ± 0.05 ^ab^	0.66 ± 0.07 ^ab^	3.04 ± 0.17 ^ab^	1.08 ± 0.21 ^b^	0.95 ± 0.01 ^ab^	1.02 ± 0.17 ^a^	0.88 ± 0.01 ^abc^	0.2 ± 0.02 ^a^	1.48 ± 0.21 ^bc^	5.04 ± 0.68 ^bc^	2.77 ± 0.28 ^bc^	1.02 ± 0.09 ^bc^	1.73 ± 0.15 ^b^	4.8 ± 0.45 ^c^
ZLP8	1.43 ± 0.13 ^ab^	0.98 ± 0.03 ^a^	0.72 ± 0.07 ^bc^	1.22 ± 0.07 ^ab^	0.65 ± 0.01 ^ab^	3.19 ± 0.28 ^ab^	1.47 ± 0.17 ^a^	0.98 ± 0.03 ^ab^	0.94 ± 0.03 ^ab^	0.81 ± 0.05 ^abc^	0.2 ± 0.07 ^a^	1.56 ± 0.15 ^bc^	5.3 ± 0.21 ^b^	3 ± 0.13 ^ab^	1.13 ± 0.15 ^ab^	1.82 ± 0.12 ^ab^	4.5 ± 0.25 ^c^
ZLP9	1.34 ± 0.05 ^abc^	0.92 ± 0.05 ^a^	0.80 ± 0.13 ^b^	1.19 ± 0.21 ^ab^	0.64 ± 0.05 ^b^	3.1 ± 0.15 ^ab^	1.16 ± 0.03 ^b^	0.92 ± 0.17 ^b^	1 ± 0.28 ^ab^	0.76 ± 0.03 ^bcd^	0.2 ± 0.03 ^a^	1.55 ± 0.17 ^bc^	5.12 ± 0.23 ^bc^	2.74 ± 0.07 ^bc^	1.06 ± 0.05 ^bc^	1.7 ± 0.21 ^b^	5.5 ± 0.41 ^a^

^a, b, c, d^: Duncan’s method was used to analyze the significant differences between the contents of the same amino acid in different samples.

**Table 3:** Flavor sensory attributes as obtained from the expert panel.

Sensory Attribute	Description
Roasted nut	A slightly oily smell reminiscent of roasted nuts.
Fresh peanuts	A smell reminiscent of raw peanuts.
Sweet aroma	A smell associated with sweetness.
Burnt	A smell reminiscent of burnt grain.
Over-burnt	A smell reminiscent of burnt or fried food.

## Data Availability

The data presented in this study are available on request from the corresponding author.

## Supplementary Materials

The following supporting information can be downloaded at: https://www.mdpi.com/article/10.3390/molecules29091947/s1, Table S1: Composition and relative content of volatile components in normal- and hi 1 gh-oleic peanut oil samples (mg/kg); Table S2: Fatty acid composition profile of different peanut oil samples (g/100 g).
