# Supplementary material for: Characterization of Key Aroma Compounds and Main Contributing Amino Acids in Hot-Pressed Oil Prepared from Various Peanut Varieties
Source: Molecules. 2024 Apr 24;29(9):1947. doi: 10.3390/molecules29091947 (PMC11085177; doi:10.3390/molecules29091947)
Supplement: Supplementary file 1 [file molecules-29-01947-s001.zip › molecules-2952813-supplementary.pdf]

Table S1. Composition and relative content of volatile components in normal- and high-oleic peanut oil samples (mg/kg).

Number	CAS	Category	Volatile Compound	Content（mg/kg）								
				ZLP1	ZLP2	ZLP3	ZLP4	ZLP5	ZLP6	ZLP7	ZLP8	ZLP9
1	74-93-1	Alcohols	Methyl mercaptan	0.261	0.192	0.189	0.195	0.153	0.186	0.105	0.12	0.108
2	71-41-0		1-Pentanol	0.519	0.372	0	0.426	0	0.405	0	0.306	0
3	111-27-3		Hexanol	0.882	1.125	0.321	1.428	1.392	1.521	0.387	1.554	1.131
4	3391-86-4		1-Octen-3-ol	0.213	0.345	0.189	0.084	0.327	0.201	0.234	0.186	0.36
5	111-87-5		Octanol	0	0	0	0	0.156	0.204	0	0.186	0.327
6	98-00-0		Furfuryl alcohol	0.885	0.594	0.633	0.441	0.786	0.552	0.564	0.507	0.57
7	143-08-8		1-Nonanol	0	0	0	0.075	0.069	0.075	0.036	0.129	0
8	100-51-6		Benzyl alcohol	0	0	0	0	0	0	0.045	0	0.075
9	1960/12/8		Phenylethanol	0	0	0.075	0.087	0.099	0.216	0.066	0.297	0.168
10	90-00-6	Phenolics	2-Ethylphenol	0	0	0	0.009	0.015	0	0	0	0.012
11	7786-61-0		4-Hydroxy-3-methoxystyrene	0.261	0.297	0.372	0.309	0.555	0.78	0.594	0.867	0.759
12	140-29-4	Others	Benzyl cyanide	0	0	0	0	0.018	0	0	0	0
13	75-50-3		Trimethylamine	0	0	0.057	0	0.051	0.039	0.117	0.117	0.192
14	75-18-3		Dimethyl sulfide	0	0	0	0.171	0	0	0	0	0
15	624-92-0		Dimethyl disulfide	0.135	0.069	0.066	0.069	0.078	0.096	0.033	0.033	0.021
16	3658-80-8		Dimethyl trisulfide	0.036	0.024	0.045	0.039	0.03	0.06	0	0.015	0
17	67-68-5		Dimethyl sulfoxide	0	0	0.078	0.255	0	0	0	0	0
18	4030-22-2		3,4-Dimethyl-3-pyrrolin-2-one	0	0.027	0.018	0.012	0.042	0	0.036	0	0
19	123-38-6	Aldehydes	Propionaldehyde	0	0	0	0	0.006	0	0.009	0	0
20	96-17-3		2-Methylbutyraldehyde	0.78	0.477	0.579	0.435	0.576	0.543	0.612	0.357	0.381
21	590-86-3		Isovaleraldehyde	0.492	0.234	0.297	0.192	0.255	0	0	0.135	0.18
22	110-62-3		Glutaraldehyde	0.291	0.219	0.126	0.12	0.162	0.123	0.111	0.099	0.156

23	66-25-1		Hexanal	1.263	1.314	0.666	0.585	1.026	0.885	0.603	0.819	0.945
24	623-36-9		2-Methyl-2-pentenal	0	0	0	0	0	0	0.018	0	0
25	111-71-7		Heptanaldehyde	0	0	0	0	0	0	0	0.303	0.462
26	6728-26-3		2-hexenal	0.084	0.12	0.045	0.03	0.126	0.099	0.051	0.048	0.123
27	124-13-0		Octanal	0.099	0.297	0.168	0.3	0.342	0.3	0.279	0.339	0.783
28	124-19-6		Nonanal	0	0	0	0	0	0	0	0	1.293
29	36431-60-4		1-Cyclopentene-1-carboxaldehyde, 5-ethyl-	0.105	0.18	0.111	0	0.207	0.162	0.114	0.099	0.219
30	1998/1/1		furfural	1.635	1.545	1.311	0.915	1.62	1.056	0.966	0.906	1.023
31	112-31-2		Decanal	0	0	0	0	0.057	0.048	0	0	0.036
32	100-52-7		Benza	0.564	0.183	0.276	0.282	0.42	0.72	0.747	0.513	0.525
33	18829-56-6		Trans-2-nonanal	0.03	0.051	0.036	0.021	0.063	0.057	0.063	0.042	0.117
34	93-53-8		2-phenylpropionaldehyde	0.036	0.036	0	0	0	0.054	0.06	0	0
35	122-78-1		Phenylacetaldehyde	0.876	0.702	0.339	0.495	0.471	1.173	1.128	1.095	0.783
36	529-20-4		2-methylbenzaldehyde	0	0	0	0	0	0	0	0.384	0
37	3913-81-3		Trans-2-decenylaldehyde	0	0	0	0	0	0	0	0	0.639
38	2463-77-6		2-undecanonal Trans-2,4-decadienal	0.036	0.087	0.051	0.069	0.108	0.108	0.09	0.126	0.24
39	2363-88-4		2,4-decadienal	0.033	0.063	0.018	0	0.117	0.102	0	0	0.168
40	25152-84-5		trans,trans-2,4-Decadien-1-al	0.108	0.228	0.102	0.099	0.399	0.381	0.3	0.195	0.591
41	4411-89-6		(2Z)-2-Phenyl-2-butenal	0.141	0.114	0.048	0.054	0.105	0.342	0.219	0.231	0.177
42	64-19-7	Acids	acetic acid	1.377	0.849	0.825	0.504	1.188	0.465	0	0.483	0.348
43	541-47-9		3,3-Dimethylacrylic acid	0.06	0.039	0.018	0.045	0	0	0.105	0	0
44	142-62-1		Caproic acid	0.321	0.456	0.318	0.471	1.002	1.326	0.399	0.996	0.972
45	124-07-2		Octanoic acid	0.051	0.093	0.09	0.078	0.108	0.21	0.051	0.219	0.249
46	112-05-0		Nonanoic acid	0.138	0.174	0.105	0.21	0.123	0.252	0.366	0.33	0.285
47	334-48-5		Capric acid	0	0	0	0	0	0.015	0	0	0

48	109-66-0	Alkanes	Pentane	0	0	0	0.006	0	0.042	0.021	0	0
49	110-54-3		hexane	0	0	0.126	0.33	0	0.084	0	0	0
50	142-82-5		Heptane	0.267	0.18	0.135	0.126	0.147	0.093	0.048	0.093	0.147
51	111-65-9		octane	0.357	0.429	0.21	0.21	0.255	0.192	0.111	0.126	0.306
52	108-88-3		toluene	0.321	0.204	0	0	0	0.174	0.165	0	0
53	1632-71-9		Nonane, 4-ethyl-5-methyl-	0	0	0	0	0	0	0.045	0.06	0
54	17302-23-7		Dimethylnonane, 2,6-	0	0.018	0.003	0	0	0.003	0	0	0
55	100-41-4		Ethyl benzene	0	0	0	0	0	0	0	0	0.003
56	108-38-3		M-xylene	0	0	0	0.006	0	0.003	0	0	0
57	112-40-3		Dodecane	0	0	0	0	0	0	0.027	0	0
58	2815-58-9		Cyclopentane, 1,2,4-trimethyl-	0	0	0.228	0	0.468	0	0.291	0	0.414
59	629-50-5		Tridecane	0	0	0.069	0	0	0	0.225	0.21	0
60	629-59-4		Tetradecane	0.033	0.075	0.039	0.03	0.057	0.054	0	0.114	0.096
61	600-14-6	Ketones	2,3-pentanedione	0.201	0.096	0.078	0.087	0.054	0.045	0.054	0.066	0.027
62	110-43-0		2-heptanone	0	0	0	0	0.099	0.075	0	0.108	0.117
63	513-86-0		3-hydroxy-2-oxobutane	0.159	0.06	0.078	0.105	0.054	0.045	0.042	0.06	0.051
64	111-13-7		Zhongoctanone	0	0	0	0	0.06	0.078	0	0.042	0.075
65	116-09-6		Hydroxyacetone	0.051	0	0	0	0	0	0	0	0
66	5704-20-1		2-hydroxy-3-pentanone	0.153	0.081	0.063	0.054	0.06	0.039	0.051	0.042	0.042
67	5077-67-8		1-hydroxy-2-butanone	0.036	0	0	0	0	0	0	0	0
68	18402-83-0		3-nonene-2-one	0	0	0	0	0.042	0.033	0	0	0.066
69	765-70-8		3-Methyl-1,2-cyclopentanedione	0.132	0	0	0.084	0	0.12	0.09	0.159	0.153
70	80-71-7		Methyl cyclopentenolone	0	0.117	0.132	0	0.189	0	0	0	0
71	3393-45-1		5,6-DIHYDRO-2H-PYRAN-2-ONE	0	0	0	0	0	0.009	0	0	0
72	6124-79-4		4-METHYL-2(5H)-FURANONE	0	0	0.021	0.012	0	0	0	0	0
73	599-04-2		2(3H)-Furanone, dihydro-3-hydroxy-4,4-dimethyl-,	0	0	0.261	0	0	0.201	0	0	0

74	96-54-8	Heterocyclics	N-Methyl pyrrole	4.005	1.551	0.42	0.558	0.849	0.267	0.585	0.534	0.507
75	289-95-2		pyrimidine	0.186	0	0	0	0	0	0	0	0
76	290-37-9		Pyrazine	0	0.087	0	0.096	0	0.054	0.057	0	0.066
77	108-99-6		3-methylpyridine	0	0	0.033	0	0	0.033	0.036	0.024	0.042
78	3777-69-3		2-Pentylfuran	0.366	0.696	0.426	0.471	1.149	0.834	0.504	0.456	1.41
79	109-08-0		2-methylpyrazine	4.812	3.195	2.13	2.511	3.372	2.436	3.039	2.238	2.316
80	123-32-0		2,5-Dimethyl pyrazine	0	4.563	2.952	3.513	5.028	4.311	4.857	4.131	4.191
81	108-50-9		2,6-Dimethyl pyrazine	1.179	1.092	0.669	0.69	1.287	0.864	1.185	0.84	0.96
82	13925-00-3		2-Ethylpyrazine	1.191	0.984	0.648	0.753	1.023	0.678	0.876	0.663	0.723
83	5910-89-4		2,3-Dimethylpyrazine	0.501	0.432	0.225	0.249	0.408	0.264	0.414	0.282	0.327
84	536-78-7		3-ethylpyridine	0	0.015	0	0	0.009	0	0	0	0.033
85	13925-03-6		2-ethyl-6-methylpyrazine	0.939	1.092	0.702	0.621	1.377	0.732	1.239	0.666	0.903
86	13360-64-0		2-ethyl-5-methylpyrazin	2.079	2.616	1.779	2.064	3.246	2.559	2.922	2.208	1.836
87	14667-55-1		2,3,5-Trimethylpyrazine	1.506	0	0	0.972	0	1.335	1.983	1.266	1.542
88	3430-33-9		3-AMINO-2,6-DIMETHYLPYRIDINE	0	1.716	0.999	0	1.98	0	0	0	0
89	18138-03-9		2-propylpyrazine	0	0	0	0.003	0	0.006	0	0	0
90	13925-07-0		3,5-Dimethyl-2-ethylpyrazine	0	0	0.117	0.111	0.198	0.126	0.183	0.09	0.165
91	13067-27-1		3-Ethyl-2,5-diMethylpyrazine	0.096	0.123	0	0	0	0	0	0	0
92	13360-65-1		3-Ethyl-2,5-diMethylpyrazine	0	2.67	2.04	0	2.742	1.965	2.502	1.941	2.418
93	15707-34-3		2,3-Dimethyl-5-ethylpyrazine	0.528	0.807	0.507	0.507	0.774	0.552	0.699	0.567	0.795
94	13925-09-2		Pyrazine, 2-ethenyl-6-methyl-	0.114	0.132	0.087	0.069	0.186	0.123	0.15	0.102	0.153
95	18138-05-1		Pyrazine, 3,5-diethyl-2-methyl-	0.342	0.174	0.375	0.267	0.426	0.285	0.411	0.255	0.366
96	1192-62-7		2-Acetylfuran	0.159	0.501	0.174	0.066	0.204	0.132	0.156	0.117	0.108
97	55138-63-1		Pyrazine, 2-methyl-5-(2-propenyl)-	0	0.171	0.114	0	0.162	0	0.279	0.15	0.201
98	636-41-9		2-Methylpyrrole	0	0.039	0	0	0.033	0	0.084	0	0
99	620-02-0		5-Methyl furfural	0.144	0.147	0.171	0.099	0.228	0.156	0.123	0.12	0.165

100	616-43-3		1H-Pyrrole, 3-methyl-	0.123	0.087	0	0	0.087	0.036	0.141	0.06	0.057
101	2294-76-0		2-pentylpyridine	0	0.042	0	0	0	0.084	0	0	0.189
102	7295-76-3		3-methoxypyridine	0	0.12	0.351	0.186	0	0	0.201	0	0
103	1192-58-1		N-methyl-2-pyrrolaldehyde	0.039	0.027	0.024	0.027	0.045	0.024	0.045	0.027	0
104	872-50-4		N-methylpyrrolidone	0.063	0	0.12	0.111	0	0.075	0.126	0.093	0
105	18217-82-8		2-Methyl-5-[(E)-1-propenyl]pyrazine	0	0	0.075	0.069	0.153	0.126	0.219	0	0.159
106	67402-83-9		1-Acetyl-1,4-dihydropyridine	0.117	0.099	0.105	0.102	0	0	0	0	0
107	497-23-4		2(5H)-Furanone	0.519	0.414	0.3	0.282	0.507	0.477	0.345	0.468	0.318
108	4030-18-6		1-acetylpyrrolidine	0	0.009	0.006	0	0.015	0.015	0	0	0.021
109	118-71-8		Methyl Maltol	0.369	0.3	0.453	0.45	0.384	0.531	0.36	0.627	0.426
110	1072-83-9		2-acetylpyrrole	0.21	0.18	0.225	0.189	0.312	0.324	0.264	0.291	0.312
111	1003-29-8		2-pyrrolaldehyde	0.18	0.165	0.102	0.096	0.195	0.21	0.171	0.231	0.219
112	616-45-5		2-pyrrolidone	0.231	0.222	0.27	0.219	0.264	0.24	0.117	0.228	0.33
113	3658-77-3		Furanone	0.537	0.402	0.315	0.327	0.468	0.444	0.456	0.585	0.39
114	3470-98-2		1-Butylpyrrolidin-2-one	0	0	0.039	0	0	0	0	0	0.051
115	28564-83-2		2,3-Dihydro-3,5-dihydroxy-6-methyl-4(H)-pyran-4-one	0.465	0.402	0.477	0.387	0.594	0.555	0	0.744	0.645
116	137-00-8		5-(2-Hydroxyethyl)-4-methylthiazole	0.102	0.081	0.132	0.156	0.102	0.216	0.12	0.21	0.126
117	496-16-2		2,3-Dihydrobenzofuran	1.62	1.467	1.899	1.485	1.902	3.087	2.7	2.112	2.058
118	67-47-0		5-Hydroxymethylfurfural	0.048	0.039	0.072	0	0.072	0.102	0	0.063	0.051
119	79-20-9	Esters	Methyl acetate	0	0	0	0.072	0.054	0.042	0	0.018	0
120	1679-47-6		alpha-Methylbutyrolactone	0.075	0	0.09	0.075	0	0	0	0.093	0
121	591-81-1		4-Hydroxybutyric acid	0	0	0	0	0	0	0.174	0	0
122	96-48-0		Gamma Butyrolactone	0.288	0.261	0.252	0.24	0.306	0.186	0	0.225	0.309
123	695-06-7		gamma-Hexalactone	0	0	0	0	0	0.108	0	0.057	0.144
124	93-60-7		Methyl nicotinate	0	0.015	0.036	0.039	0.057	0.051	0.057	0.072	0.045
125	104-50-7		Propionolactone	0	0	0	0	0	0.045	0	0	0

126	13129-23-2	Methyl 3-furanoate	0	0	0	0.006	0.024	0	0	0	0
127	79-50-5	DL-Pantolactone	0.276	0.213	0	0.309	0.243	0	0.207	0.27	0.237

Table S2. Fatty acid composition profile of different peanut oil samples (g/100 g).

	C14:0	C16:0	C16:1	C17:0	C18:0	C18:1n9c	C18:2n6c	C20:0	C20:1	C18:3n3	C21:0	C20:2	C22:0	C22:1n9	C23:0	C24:0	C20:5
ZLP1	0.04%	10.27%	0.06%	0.09%	2.14%	43.57%	36.13%	1.21%	1.49%	0.09%	0.02%	0.05%	2.97%	0.14%	0.04%	1.63%	0.06%
ZLP2	0.05%	12.31%	0.11%	0.09%	4.39%	50.32%	25.08%	1.74%	0.87%	0.05%	0.03%	/	3.37%	0.06%	0.05%	1.37%	0.12%
ZLP3	0.04%	9.29%	0.09%	0.08%	3.35%	54.69%	25.28%	1.48%	1.28%	0.09%	/	0.03%	2.61%	0.10%	0.05%	1.55%	/
ZLP4	0.04%	9.18%	0.09%	0.08%	3.85%	62.56%	17.53%	1.62%	1.02%	0.06%	/	0.03%	2.50%	0.07%	0.04%	1.32%	/
ZLP5	0.05%	12.31%	0.11%	0.09%	4.39%	50.32%	25.08%	1.74%	0.87%	0.05%	0.03%	/	3.37%	0.06%	0.05%	1.37%	0.12%
ZLP6	0.05%	11.08%	0.05%	0.08%	5.54%	41.55%	34.30%	2.04%	0.69%	0.07%	0.03%	0.03%	3.18%	0.05%	0.05%	1.16%	0.04%
ZLP7	0.04%	10.17%	0.05%	0.08%	5.79%	42.18%	35.13%	2.08%	0.63%	0.06%	0.02%	0.02%	2.64%	0.04%	0.04%	1.02%	/
ZLP8	0.05%	9.97%	0.07%	0.11%	3.14%	55.14%	24.04%	1.48%	1.15%	0.07%	0.03%	/	3.11%	0.09%	0.04%	1.46%	0.05%
ZLP9	0.04%	9.55%	0.07%	0.09%	2.91%	53.89%	26.04%	1.40%	1.29%	0.08%	/	0.03%	2.97%	0.11%	0.05%	1.46%	/
